# Antiangiogenic Therapy for Malignant Brain Tumors: Does It Still Matter?

**DOI:** 10.1007/s11912-023-01417-1

**Published:** 2023-04-18

**Authors:** Alessia Pellerino, Francesco Bruno, Riccardo Soffietti, Roberta Rudà

**Affiliations:** Division of Neuro‑Oncology, Department of Neuroscience “Rita Levi Montalcini”, University and City of Health and Science Hospital, 10126 Turin, Italy

**Keywords:** Antiangiogenic therapy, Bevacizumab, Glioblastoma, High-grade glioma, Tyrosine kinase inhibitors

## Abstract

**Purpose of Review:**

To summarize the mechanisms of tumor angiogenesis and resistance to antiangiogenic therapy, and the influence on tumor microenvironment.

**Recent Findings:**

Several clinical trials have investigated the activity of anti-VEGF monoclonal antibodies and tyrosine kinase inhibitors in glioblastoma, shedding the light on their limitations in terms of disease control and survival. We have outlined the mechanisms of resistance to antiangiogenic therapy, including vessel co-option, hypoxic signaling in response to vessel destruction, modulation of glioma stem cells, and trafficking of tumor-associated macrophages in tumor microenvironment. Moreover, novel generation of antiangiogenic compounds for glioblastoma, including small interfering RNAs and nanoparticles, as a delivery vehicle, could enhance selectivity and reduce side effects of treatments.

**Summary:**

There is still a rationale for the use of antiangiogenic therapy, but a better understanding of vascular co-option, vascular mimicry, and dynamic relationships between immunosuppressive microenvironment and blood vessel destruction is crucial to develop next-generation antiangiogenic compounds.

## Introduction

Angiogenesis is the growth of new blood vessels, which is typical of high-grade gliomas (HGG) and glioblastomas (GBM), the most common primary malignant brain tumors in adults. The heterogeneous histopathologic appearance of GBM includes extensive proliferation of endothelial cells (EC), with glomeruloid vessel–like structures, that are supported by basal lamina and pericytes with a lack of astrocytic end-feets. Thus, given the poor prognosis of GBM following surgery and radiotherapy with concomitant and adjuvant chemotherapy with temozolomide [1, 2], antiangiogenic therapy has been the most investigated strategy for GBM in the last decade. In this regard, the human monoclonal antibody (mAb) bevacizumab (Bev), that targets vascular endothelial growth factor (VEGF)-A, achieved the approval by the US Food and Drug Administration (FDA) for the treatment of GBM at first relapse after the standard chemoradiation based on the prolonged progression-free survival (PFS) and clinical benefit, such as relief of neurological symptom and reduction of steroids [3–5]. However, Bev did not prolong overall survival (OS) in patients with newly diagnosed or recurrent GBM in phase 3 clinical trials [5–7]. Nevertheless, a rationale for targeting neoangiogenesis still matters, since angiogenesis influences the immunosuppressive tumor microenvironment (TME) in GBM. Hence, reducing the angiogenic pathways in the TME could increase antitumor immune response. On the other hand, the significant reduction of tumor vasculature following antiangiogenic therapy causes hypoxia, leading to activation of alternative pathways to maintain tumor angiogenesis [8, 9]. Moreover, glioma neoangiogenesis results in tortuous blood vessels, that interfere with the blood–brain barrier (BBB) permeability [10], resulting in unequal drug distributions into brain tumors [11, 12].

## Angiogenesis and Mechanisms of Resistance to Antiangiogenic Therapy

Several cellular and molecular mechanisms are involved in tumor angiogenesis. The rapid tumor proliferation causes severe hypoxia and nutrient deprivation, and enhances the production of angiogenic cytokines and matrix metalloproteinases (MMP) by TME, causing an activation of EC, pericytes, reactive astrocytes, tumor-associated macrophages (TAM), and neoplastic cells. This pro-angiogenic and pro-inflammatory TME favors the formation of leaky and abnormal blood vessels, that are not able to efficiently deliver nutrients, oxygen, and drugs.

Glioma stem cells (GSC) create tube-like structures devoided of vascular EC and containing red blood cells, therefore known as vasculogenic mimicry (VM). These vascular structures may merge with micro-vessels formed by angiogenesis to support blood and nutrient supply, as well as favor the passage of glioma cells directly into the bloodstream. Different key players from TME trigger the VM, such as HIF1a, epithelial-mesenchymal transition (EMT), and VE-cadherin/EphA2/MMP signaling pathways, that enhance the hypoxic environment, while adenosine/STAT3/IL-6 pathway, MAPK/ERK pathway, Wnt/b-catenin, Notch, Wnt, Hedgehog, and Hippo signaling pathway are primarily involved in increasing the GSC pool [13, 14].

Pro-angiogenic and pro-inflammatory cytokines, including HIF1a, VEGF, IL6, and CX3CL1, induce the infiltration and differentiation of bone marrow–derived mesenchymal cells (BM-MC) into macrophages and pericytes, that modulate the balance between pro- and antiangiogenic cytokine production, and enhance the EC survival.

Activation of COX2 results in an overexpression of prostaglandin E2 (PGE2), thromboxane A2 (TXA2), and prostaglandin I2 (PGI2), that increases migration, sprouting, and proliferation of EC and glioma angiogenesis. Notably, the interaction of epidermal growth factor receptors (EGFR) with signal transducer and activator of transcription 3 (STAT3) or the constitutive activation of EGFR variant III/STAT3 pathway enhances the COX2 signaling and favors glioma angiogenesis.

Overexpression of some tyrosine kinase receptors is involved in glioma angiogenesis, such as VEGF receptors (VEGFR), platelet-derived growth factor receptors (PDGFR), and Eph receptors. VEGFR-2 and VEGFR-3 guide neoangiogenesis for blood and lymphatic vessels, respectively, while VEGFR-1 inhibits neoangiogenesis. The PDGFR regulate pericytes and smooth muscle cells activity to stabilize vascular wall, and Eph receptor defines arterial or venous identity. Additionally, the overexpression of mitogen-activated protein kinase (MAPK) and phosphoinositide 3-kinase (PI3K)-Akt pathways supports angiogenesis, tumor proliferation, and escape from apoptosis [15].

Although targeted therapy with mAbs or tyrosine kinase inhibitors (TKIs) may provide a transient period of normalization of tumor vasculature, different mechanisms of resistance to antiangiogenic therapy have been identified. Compensatory angiogenic signaling is activated by means of HIF1a, Notch, and Ang2/Tie2 signaling pathways within the hypoxic GBM TME. An immunological escape may occur: monocytes, TAM, reactive astrocytes, myeloid cells, neutrophils, and T helper-17 cytokines promote the infiltration of the pro-angiogenic clones of BM-MC resulting in tumor angiogenesis.

An increased pericyte coverage after antiangiogenic therapy contributes to sustain the survival of EC. Vessel co-option consists in the migration of tumor cells toward and along the preexisting vasculature. Typically, a subset of GSC with pericyte differentiation under the production of bradykinin/bradykinin receptor-2 (B2R), CXCR4/SDF-1a, MDGI/FABP3, EGFRvIII, and Olig2/Wnt7a increase pericyte coverage in the co-opted blood vessels and support survival of EC by promoting an autocrine VEGF-A signaling pathway [16, 17]. Autophagy provides energy for neoplastic cells to survive under hypoxic conditions through HIF-1-dependent mechanisms.

## Clinical Trials on Antiangiogenic Monoclonal Antibodies (mAbs) in GBM

Overall, the main aim of antiangiogenic treatment for GBM is to regulate the permeability of tumor vasculature and reduce the growth of new tumor vessels. When the permeability of BBB is normalized, a more effective delivery of antineoplastic compounds with adequate CNS concentrations should be achieved. An ideal antiangiogenic drug should meet some characteristics, such as high selectivity, targeting multiple signaling pathways, low risk of drug-induced resistance, enhancing the production of endogenous antiangiogenetic molecules, and limited systemic toxicity.

Antiangiogenic mAbs have been investigated in several clinical trials, showing some activity in terms of PFS, but disappointing results regarding OS. Bev, a human monoclonal antibody, which binds circulating VEGF-A, conferred an advantage in 6-month PFS when associated with irinotecan (50%) as compared with Bev alone (42%) in the BRAIN trial [3], leading to the approval by FDA for the use at first recurrence. Similarly, in Europe, the BELOB trial has shown encouraging results for the combination of Bev and lomustine versus either agent alone [18]. However, the phase 3 trial investigating the combination of Bev plus lomustine in comparison with lomustine alone failed to demonstrate an improvement of OS (median OS 9.1 months versus 8.6 months) despite an increase of PFS from 1.5 to 4.2 months [5]. Other phase 2 trials have investigated Bev in association with several drugs, including temozolomide, fotemustine, irinotecan, temsirolimus, and erlotinib, but none has displayed a significant impact on OS [19–22]. Although some concerns regarding fertility arise when Bev is used in patients with GBM at childbearing ages, in general it is well tolerated and serious adverse events, such as gastrointestinal perforation, thromboembolic events, renal injury, impairment of wound healing process, posterior reversible encephalopathy syndrome, congestive heart failure, and uncontrolled hypertension, are rare. A post hoc analysis of the ARTE trial has shown a survival benefit from the addition of Bev to radiotherapy in comparison with Bev alone in elderly patients with newly diagnosed GBM. This effect could depend on the presence of large contrast-enhancing lesions, while the detection of non-contrast-enhancing tumor on amino acid PET scans was associated with inferior survival. These findings suggest that Bev may work as a radiosensitizer in presence of dysfunctional vasculature in GBM [23, 24]. Some preclinical and translational studies have displayed that the effect of VEGF-targeted therapy on tumor barrier permeability is transient and dose-dependent [25, 26]. Notably, lower doses (< 10 mg/kg) of Bev may induce reduction of leakiness, improve oxygenation without inducing vessel destruction, and favor the up-regulation of angiopietin-2 (Ang-2), a potent driver of vessel leakiness [27]. Hence, low dose of VEGF-targeted therapy in association with Ang-2-targeted treatment has been proposed to overcome resistance to antiangiogenic therapies by regulating GBM barrier permeability and modulating the pro-tumorigenic effects of endothelial cell destruction [28]. The Ang-2 neutralizing antibody MEDI3617 was evaluated in combination with Bev in a phase 1b study in 116 patients with solid tumors, including 13 GBM patients, but unfortunately showed a poor activity (0% of radiological response in GBM) [29].

Aflibercept is a recombinant human fusion protein, that acts as a soluble decoy receptor for VEGF-A, VEGF-B, and placental growth factor, thus depleting circulating levels of these growth factors. A phase 1 trial suggested that aflibercept in combination with temozolomide could confer moderate toxicities, including fatigue, hypertension, lymphopenia, ischemic stroke, and systemic hemorrhage. All patients stopped the treatment: 28 (47%) for disease progression, 21 (36%) for toxicities, 8 (14%) for other reasons, and 2 (3%) patients only completed the full treatment course [30]. The phase 2 trial reported limited efficacy of aflibercept in both grade 3 astrocytomas (radiological response in 44%, 6-month PFS of 25%, median PFS of 24 weeks) and GBM (radiological response in 18%, 6-month PFS of 7.7%, median PFS of 12 weeks) [31].

Tanibirumab is a fully human monoclonal antibody targeting soluble VEGFR-2, that was investigated in a phase 2 trial in 12 patients with recurrent GBM. The best radiological response was a stable disease in 3/12 (25%) patients of whom 2 patients had a long-lasting response of 60 and 40 weeks, respectively, and was correlated with the highest expression of VEGFR2 using immunohistochemistry on archival tumor [32].

Preclinical data on targeting VEGFR2 are emerging. Chen et al. have shown that the anti-VEGFR2 mAb MSB0254 inhibits the invasion and migration of U251 and primary glioma cells in vitro. Moreover, MSB0254 also significantly inhibits the expression of CD34, VEGFR2, Ki67, MMP2, and MMP9 and reduces the VM formation, resulting a compound to be further investigated for the treatment of GBM [33•].

As VEGF-targeted mAbs have failed to control disease in GBM, we argue that vascular normalization is not the sole factor to overcome treatment resistance of GBM, and other mechanisms may co-exist or even prevail upon targeting neoangiogenesis.

## Targeting Vessel Co-option

Wnt-7 has been identified as a driver of vessel co-option in a subpopulation of GSC with features of oligodendrocyte precursor cells. Wnt-7 is secreted by the membrane-bound O-acyl transferase porcupine, that can be targeted by the BBB penetrating small molecule inhibitor LGK974. In a glioblastoma xenograft model, LGK974 reduced vessel co-option and VEGF expression, but data on clinical activity on human GBM, as well as the ability to cross the BBB, are lacking [34••, 35]. CXCR-4 positive GSC are up-regulated following Bev [36] and can be targeted by the small molecular CXCR4 inhibitor, plerixafor. In a phase 1 trial of plerixafor with Bev in patients with recurrent HGG, remarkable concentrations of the small molecular inhibitor were identified in the CSF and brain tumor tissue, as well as biomarker changes consistent with VEGF and CXCR-4 inhibition. Unfortunately, despite the demonstration of adequate drug penetration and downstream effects, the efficacy of this combination in disease control was limited [37]. The mammalian target of rapamycin (mTOR) promotes anabolic metabolism of GSC, invasiveness, and poor sensitivity to chemo- and radiotherapy [38]. However, the mTOR inhibitor temsirolimus failed to prolong OS when associated with radiotherapy versus radiotherapy plus temozolomide in patients with newly diagnosed unmethylated O6-methylguanine-O-methyl-transferase (MGMT) GBM. Interestingly, a small subgroup of patients with Ser2448 phosphorylation of mTOR derived a strong benefit from temsirolimus [39]. Whether temsirolimus may overcome resistance to Bev by specifically targeting GSC has not been studied thus far.

In summary, some preclinical studies suggest that targeting vessel co-option could provide a synergic activity with antiangiogenic therapy, but efficacy of such an approach is far to be demonstrated.

## Co-regulation of Angiogenesis and Immunosuppressive TME in GBM

Tumor microenvironment of malignant gliomas is immunologically “cold” as it is dominated by immunosuppressive and pro-angiogenic cells, including 80% of macrophages, monocytes, and neutrophils, with < 10% of dendritic cells, T cells, and natural killer cells [40]. Furthermore, tumor-derived soluble factors contribute to the immunosuppressive microenvironment [41•]. In this regard, interferon (IFN)-γ or lipopolysaccharide promotes a pro-inflammatory phenotype of macrophages (phagocytic “M1”), while autocrine and paracrine stimulation by IL-4, IL-6, IL-10, or TGF-β induce an immunosuppressive “M2” phenotype of macrophages. Importantly, hypoxia and HIF-1 favor M2-polarization of perinecrotic macrophages in experimental gliomas [42, 43]. VEGF exerts an immunosuppressive activity by inducing down-regulation of antigen presentation through the inhibition of dendritic cell maturation: involved mechanisms are the inhibition of nuclear factor-κB (NF-κB), and up-regulation of PD-L1 on myeloid and endothelial cells, that lead to inhibition of T cell extravasation and activation, inhibition of T cell differentiation in the thymus, expansion of inhibitory regulatory T cells (Treg), and inhibition of cytotoxic CD8 + T effector cells, such as PD-1, CTLA-4, T cell immunoglobulin mucin receptor 3 (TIM3), and lymphocyte activation gene 3 protein (LAG3).

TGF-β has been considered a master of immunosuppression of TME, but inhibiting TGF-β signaling with the targeted therapy with galunisertib was unsuccessful in a phase 2 clinical trial in patients with GBM [44]. Moreover, the combination of galunisertib with anti-VEGF treatment did not confer any significant benefit [45].

VEGF and hypoxia can drive the expression of chemoattractants, such as the CC-chemokine ligand 2 (CCL-2), via CC-chemokine receptor 2 (CCR2) and CCR4, leading to a higher level of CSF-1, that stimulates a subpopulation of pro-angiogenic macrophages expressing the Ang-2 receptor Tie-2, and co-opts micro-vessels and enhances M2 macrophages [46, 47]. The small molecule CSF-1 R inhibitor BLZ945, which is a CSF-1 R inhibitor, can enhance a pro-inflammatory M1 phenotype of macrophages and prolong survival in platelet-derived growth factor (PDGF)–driven genetic glioblastoma models, as well as reduce VEGF-driven proliferation of macrophages. In this regard, a phase 1/2 trial of BLZ945 in association with the PD-1 antibody spartalizumab in solid tumors, including GBM, has completed the enrollment in December 2022, and the analyses for the primary outcomes (dose-limiting toxicities, maximum tolerated dose, incidence of adverse events, and PFS) are ongoing (NCT02829723).

The low mutational burden and the immunosuppressive TME explain the failure of anti-PD1 compounds in phase 3 clinical trials on GBM [48]. However, some lymphocyte-independent macrophages can be stimulated by targeting the PD-1 pathway [49], and be active also in genetic glioblastoma models with low antigen expression [50]. Notably, the use of an oncolytic virus, designed to reprogram macrophages, induced an up-regulation of VEGF; thus, the administration of a VEGF antibody enhanced the antitumor activity of viral therapy [51]. Similar results were reported by Saha et al. who demonstrated that T cell–independent cooperation can be increased by a viral therapy in combination with the VEGF inhibitor axitinib in a transplantation-based hypoimmunogenic glioblastoma model [52], supporting the concept that targeting VEGF can support macrophage repolarization.

Although preclinical and clinical studies have shown the feasibility of chimeric antigen receptors (CAR) T cell immunotherapeutic approach in GBM, tumor heterogeneity, and antigen loss remain one of the upmost challenges to be addressed. Rousso-Noori et al. have identified the p32/gC1qR/HABP/C1qBP as a specific tumor-associated antigen expressed on the surface of glioma cells, resulting a feasible target for CAR T cell therapy with ability to control tumor growth in orthotopic syngeneic and xenograft mouse models. Therefore, further investigation on such a dual antitumor and antiangiogenic p32 CAR T cells will be warranted [53••].

## Vascular Characteristics Among Glioma Subtypes

Proneural GBM has been reported to express a predominance of vascular co-option [34••], while mesenchymal GBM has a higher abundance of macrophages, vascular abnormalities, hypoxia, and necrosis [54]. The post hoc analyses of the phase 3 AVAglio trial on radiation plus temozolomide with versus without Bev reported an OS advantage for the proneural GBM [55]; however, such an association was not confirmed in other studies [23, 56].

The vasculature of IDH-mutated GBM has a lower frequency of vascular abnormalities and necrosis as compared with IDH wild-type GBM [57]. The F3 gene, which encodes the key prothrombotic protein tissue factor, is downregulated in IDH-mutated GBM [58], resulting in an increased cerebral blood flow [59], reduced angiogenesis, and abnormal tumor vasculature due to the inhibition of mesenchymal GSC phenotypes [60]. Furthermore, IDH-mutated GBM escape from adaptive immunity by down-regulating major histocompatibility complex I to prevent antigen presentation by tumor cells [61], and suppressing chemotaxis-associated gene expression programs for T cell activity [62]. IDH inhibitors stimulate T cell infiltration and activity of a peptide vaccine against IDH-mutated gliomas in vivo [62, 63]: however, the influence on the altered vasculature remains unknown. In a phase 2 clinical trial of Bev in combination with temozolomide compared to temozolomide alone in IDH-mutated astrocytic brain tumors, no efficacy of Bev was observed [64].

## Clinical Trials of Antiangiogenic Tyrosine Kinase Inhibitors in GBM

Axitinib is an oral small multi-TKIs targeting VEGF1-3, c-KIT, and PDGFR, that was investigated in different clinical trials, showing frequent grade 3/4 adverse events, including fatigue, diarrhea, and oral hyperesthesia. In a phase 2 trial in recurrent GBM, axitinib showed an ORR and 6-month PFS of 28% and 34%, respectively, as compared with 23% and 28% of patients treated with Bev or lomustine [65]. Another phase 2 trial has investigated whether the association of axitinib and lomustine could improve ORR and PFS in recurrent GBM, but the combination therapy did not show any advantage compared with axitinib alone (ORR 38%, 6-month PFS 17%, OS 27.4 weeks in the axitinib plus lomustine arm; ORR 28%, 6-month PFS 26%, OS 29 weeks in the axitinib arm) [66]. Unfavorable results of another phase 2 trial on the efficacy and tolerability of axitinib plus avelumab led to a discontinuation of further investigations [67].

Cabozantinib is a multi-kinase inhibitor of VEGFR2, c-MET, AXL, and RET, which was evaluated in a phase 1 trial in association with chemoradiation in newly diagnosed GBM, showing a manageable profile [68], and is under investigation in association with the anti-PD-L1 atezolizumab in a phase 1/2 clinical trial on recurrent GBM (NCT05039281).

Nintedanib is an oral, small-molecule TKI of PDGFR α/β, FGFR 1–3, and VEGFR 1–3, that may overcome resistance to anti-VEGF therapy. Although nintedanib was well tolerated, two different trials did not display any activity against recurrent GBM regardless of prior Bev therapy [69, 70].

Regorafenib is an oral inhibitor of several kinases involved in tumor angiogenesis (VEGFR1-3 and TIE2), oncogenesis (KIT, RET, RAF1, and BRAF), and in the interaction between tumor and microenvironment (PDGFR, FGFR), and tumor immunity (colony-stimulating factor 1 receptor [CSF1R]). In the randomized, open-label, phase 2 REGOMA trial, GBM patients at first recurrence were treated with either regorafenib or lomustine displaying an OS of 7.4 vs 5.6 months, as well as 6-month PFS of 16.9% vs 8.3%, respectively [71]. Of note, the OS of patients treated with lomustine from REGOMA trial was remarkably short (5.6 months) as compared with that of patients included in the lomustine single arms of other randomized controlled trials (median OS 7.1–10.4 months [5, 72, 73], which could imply an overestimation of regorafenib efficacy. Few other studies have shown similar impact on survival [74–79], but a higher rate of adverse events than in REGOMA, thus raising concerns over tolerability. A lower intensity regimen proved as effective as the standard 160 mg daily schedule used in REGOMA trial (median PFS and median OS of 2.0 months and 7.4 months), but with lower adverse events [80]. The AGILE trial (NCT03970447) will help to clarify the role of regorafenib in patients with newly diagnosed GBM without MGMT promoter methylation.

Other VEGF multi-kinase inhibitors have been evaluated in recurrent GBM, such as sunitinib, sorafenib, ponatinib, and vandetanib, and all showed minimal or absent activity or raised major concerns for serious adverse events [81•].

## Conclusions

Angiogenesis is a crucial mechanism for tumor cell survival, providing nutrients and oxygen, and promotes tumor immunosuppression. Significant efforts have been made to develop and evaluate the efficacy of novel antiangiogenic drugs for HGG, especially mAbs and drugs. The efficacy of an anti-VEGF mAb presents different barriers, ranging from low penetration into tumor tissue to failure to adequately cross the BBB due to the large size of the compounds. Conversely, TKIs have smaller size and target angiogenesis via different pathways but have low selectivity resulting in major systemic toxicities, as well as increased risk of acquired resistance.

Next-generation antiangiogenic therapies aim to overcome these limits. In this regard, small interfering RNA (siRNAs) are potent effective silencers of tumor angiogenic gene expression in GBM when loaded in tumor-targeted nanoparticles. These compounds display several advantages, including minimal recognition by the immune system, blood stability, high specificity, and low systemic side effects [82•]. Hence, nanotechnology is working on the development of novel delivery systems that can improve delivery of siRNAs and protect them from degradation and systemic clearance. To date, two different studies have explored carriers for siRNA delivery in GBM. A phase 1 study examined the side effects and best dose of DOPC-encapsulated EphA2 siRNA in the treatment of patients with metastatic solid tumors or recurrent GBM and demonstrated that this compound is able to slow the growth of tumor cells (NCT01591356). Another phase 0 study has evaluated a potential treatment for GBM with the use of RNA-interfering spherical nucleic acids (SNAs), that consist of nuclei of gold nanoparticles covalently bonded to Bcl2L12 siRNA oligonucleotides, that can penetrate the brain. In this study, patients with recurrent GBM were treated with intravenous administration of siBcl2L12-SNAs revealing remarkable gold enrichment in the tumor-associated endothelium, macrophages, and tumor cells, as well as reduction in tumor-associated Bcl2L12 protein expression [83••]. Overall, numerous nanoplexes are being tested in preclinical setting, and could serve as potential next-generation antiangiogenic therapeutics for GBM [84–88].

Lastly, it is unclear whether and how antiangiogenic therapy and immunotherapy should be combined with radiotherapy and/or chemotherapy. Given that radiotherapy may favor antigen release from tumor cells, apoptosis of EC, and promote the influx of monocytes into TME, the combinations with immunotherapy and antiangiogenic therapy may enhance this effect and should be evaluated preclinically in the context of current standard treatments.

## References

[1] Stupp R, Mason WP, van den Bent MJ, Weller M, Fisher B, Taphoorn MJ (2005). Radiotherapy plus concomitant and adjuvant temozolomide for glioblastoma. N Engl J Med.

[2] Perry JR, Laperriere N, O'Callaghan CJ, Brandes AA, Menten J, Phillips C (2017). Short-course radiation plus temozolomide in elderly patients with glioblastoma. N Engl J Med.

[3] Friedman HS, Prados MD, Wen PY, Mikkelsen T, Schiff D, Abrey LE (2009). Bevacizumab alone and in combination with irinotecan in recurrent glioblastoma. J Clin Oncol.

[4] Kreisl TN, Kim L, Moore K, Duic P, Royce C, Stroud I (2009). Phase II trial of single-agent bevacizumab followed by bevacizumab plus irinotecan at tumor progression in recurrent glioblastoma. J Clin Oncol.

[5] Wick W, Gorlia T, Bendszus M, Taphoorn M, Sahm F, Harting I (2017). Lomustine and bevacizumab in progressive glioblastoma. N Engl J Med.

[6] Chinot OL, Wick W, Mason W, Henriksson R, Saran F, Nishikawa R (2014). Bevacizumab plus radiotherapy-temozolomide for newly diagnosed glioblastoma. N Engl J Med.

[7] Gilbert MR, Dignam JJ, Armstrong TS, Wefel JS, Blumenthal DT, Vogelbaum MA (2014). A randomized trial of bevacizumab for newly diagnosed glioblastoma. N Engl J Med.

[8] Bulnes S, Bengoetxea H, Ortuzar N, Argandoña E.G, Garcia-Blanco A, Rico-Barrio I, et al. Angiogenic signalling pathways altered in gliomas selection mechanisms for more aggressive neoplastic subpopulations with invasive phenotype J Signal Transduct 2012 597915 10.1155/2012/597915. 10.1155/2012/597915 PMC3407647 22852079

[9] Hu Q, Liu F, Yan T, Wu M, Ye M, Shi G (2019). MicroRNA-576-3p inhibits the migration and proangiogenic abilities of hypoxia-treated glioma cells through hypoxia-inducible factor-1α Int. J Mol Med.

[10] Liebner S, Dijkhuizen RM, Reiss Y, Plate KH, Agalliu D, Constantin G (2018). Functional morphology of the blood-brain barrier in health and disease. Acta Neuropathol.

[11] Arvanitis CD, Ferraro GB, Jain RK (2020). The blood-brain barrier and blood-tumour barrier in brain tumours and metastases. Nat Rev Cancer.

[12] Mo F, Pellerino A, Soffietti R, Rudà R (2021). Blood-brain barrier in brain tumors: biology and clinical relevance. Int J Mol Sci.

[13] Xiang T, Lin YX, Ma W, Zhang HJ, Chen KM, He GP (2018). Vasculogenic mimicry formation in EBV-associated epithelial malignancies. Nat Commun.

[14] Wei X, Chen Y, Jiang X, Peng M, Liu Y, Mo Y (2021). Mechanisms of vasculogenic mimicry in hypoxic tumor microenvironments. Mol Cancer.

[15] Kim G, Ko YT (2020). Small molecule tyrosine kinase inhibitors in glioblastoma. Arch Pharm Res.

[16] Seano G, Jain RK (2020). Vessel co-option in glioblastoma: emerging insights and opportunities. Angiogenesis.

[17] Kuczynski EA, Reynolds AR (2020). Vessel co-option and resistance to anti-angiogenic therapy. Angiogenesis.

[18] Taal W, Oosterkamp HM, Walenkamp AM, Dubbink HJ, Beerepoot LV, Hanse MC (2014). Single-agent bevacizumab or lomustine versus a combination of bevacizumab plus lomustine in patients with recurrent glioblastoma (BELOB trial): a randomised controlled phase 2 trial. Lancet Oncol.

[19] Raizer JJ, Giglio P, Hu J, Groves M, Merrell R, Conrad C (2016). Brain tumor trials collaborative A phase II study of bevacizumab and erlotinib after radiation and temozolomide in MGMT unmethylated GBM patients. J Neurooncol.

[20] Lassen U, Sorensen M, Gaziel TB, Hasselbalch B, Poulsen HS (2013). Phase II study of bevacizumab and temsirolimus combination therapy for recurrent glioblastoma multiforme. Anticancer Res.

[21] Soffietti R, Trevisan E, Bertero L, Cassoni P, Morra I, Fabrini MG (2014). Bevacizumab and fotemustine for recurrent glioblastoma: a phase II study of AINO (Italian Association of Neuro-Oncology). J Neurooncol.

[22] Gilbert MR, Pugh SL, Aldape K, Sorensen AG, Mikkelsen T, Penas-Prado M (2017). NRG oncology RTOG 0625: a randomized phase II trial of bevacizumab with either irinotecan or dose-dense temozolomide in recurrent glioblastoma. J Neurooncol.

[23] Wirsching HG, Tabatabai G, Roelcke U (2018). Bevacizumab plus hypofractionated radiotherapy versus radiotherapy alone in elderly patients with glioblastoma: the randomized, open-label, phase II ARTE trial. Ann Oncol.

[24] Wirsching HG, Roelcke U, Weller J (2020). MRI and 18FET-PET predict survival benefit from bevacizumab plus radiotherapy in patients with IDH wild-type glioblastoma: results from the randomized ARTE trial. Clin Cancer Res.

[25] Batchelor TT, Sorensen AG, di Tomaso E, Zhang WT, Duda DG, Cohen KS (2007). AZD2171, a pan-VEGF receptor tyrosine kinase inhibitor, normalizes tumor vasculature and alleviates edema in glioblastoma patients. Cancer Cell.

[26] Emblem KE, Mouridsen K, Bjornerud A, Farrar CT, Jennings D, Borra RJ (2013). Vessel architectural imaging identifies cancer patient responders to anti-angiogenic therapy. Nat Med.

[27] Kloepper J, Riedemann L, Amoozgar Z, Seano G, Susek K, Yu V (2016). Ang2/VEGF bispecific antibody reprograms macrophages and resident microglia to anti-tumor phenotype and prolongs glioblastoma survival. Proc Natl Acad Sci U S A.

[28] Peterson TE, Kirkpatrick ND, Huang Y, Farrar CT, Marijt KA, Kloepper J (2016). Dual inhibition of Ang-2 and VEGF receptors normalizes tumor vasculature and prolongs survival in glioblastoma by altering macrophages. Proc Natl Acad Sci U S A.

[29] Hyman DM, Rizvi N, Natale R, Armstrong DK, Birrer M, Recht L (2018). Phase I study of MEDI3617, a selective angiopoietin-2 inhibitor alone and combined with carboplatin/paclitaxel, paclitaxel, or bevacizumab for advanced solid tumors. Clin Cancer Res.

[30] Groot J, Wefel JS, Cloughesy TF, Lieberman F, Chang SM (2017). Phase I trial of aflibercept (Vegf trap) with radiation therapy and concomitant and adjuvant temozolomide in patients with high-grade gliomas. J Neurooncol.

[31] de Groot JF, Lamborn KR, Chang SM, Gilbert MR, Cloughesy TF, Aldape K (2011). Phase II study of aflibercept in recurrent malignant glioma: a North American Brain Tumor Consortium study. J Clin Oncol.

[32] Cher L, Nowak A, Iatropoulos G, Lee WS, Lee SY, Shim SR, et al. ACTR-75. A multicenter 3-arm open-label, phase 2a clinical trial to evaluate safety and efficacy of tanibirumab (VEGFR2 MAB) in patients with recurrent GBM assessed with K-trans and initial area under the gadolinium concentration-time curve (IUGC) Neuro Oncol. 2017;19(Suppl 6):vi17.

[33] • Chen S, Li X, Wang H, Chen G, Zhou Y. Anti-VEGFR2 monoclonal antibody (MSB0254) inhibits angiogenesis and tumor growth by blocking the signaling pathway mediated by VEGFR2 in glioblastoma. Biochem Biophys Res Commun. 2022;604:158-164. **A novel anti-VEGFR2 monoclonal antibody with promising results both in vitro and in vivo analyses.** 10.1016/j.bbrc.2022.03.045 35305420

[34] •• Griveau A, Seano G, Shelton SJ, Kupp R, Jahangiri A, Obernier K, et al. A glial signature and Wnt7 signaling regulate glioma-vascular interactions and tumor microenvironment. Cancer Cell. 2018;33(5):874-889.e7. **This study establishes vessel co-option as a mechanism of resistance to antiangiogenic agents employed by GSC.** 10.1016/j.ccell.2018.03.020 PMC6211172 29681511

[35] Jung YS, Park JI (2020). Wnt signaling in cancer: therapeutic targeting of Wnt signaling beyond β-catenin and the destruction complex. Exp Mol Med.

[36] Pham K, Luo D, Siemann DW, Law BK, Reynolds BA, Hothi P (2015). VEGFR inhibitors upregulate CXCR4 in VEGF receptor-expressing glioblastoma in a TGFβR signaling-dependent manner. Cancer Lett.

[37] Lee EQ, Duda DG, Muzikansky A, Gerstner ER, Kuhn JG, Reardon DA (2018). Phase I and biomarker study of plerixafor and bevacizumab in recurrent high-grade glioma. Clin Cancer Res.

[38] Kumar S, Sharife H, Kreisel T, Mogilevsky M, Bar-Lev L, Grunewald M (2019). Intra-tumoral metabolic zonation and resultant phenotypic diversification are dictated by blood vessel proximity. Cell Metab.

[39] Wick W, Gorlia T, Bady P, Platten M, van den Bent MJ, Taphoorn MJ (2016). Phase II study of radiotherapy and temsirolimus versus radiochemotherapy with temozolomide in patients with newly diagnosed glioblastoma without MGMT promoter hypermethylation (EORTC 26082). Clin Cancer Res.

[40] Friebel E, Kapolou K, Unger S, Núñez NG, Utz S, Rushing EJ (2020). Single-cell mapping of human brain cancer reveals tumor-specific instruction of tissue-invading leukocytes. Cell.

[41] • Chongsathidkiet P, Jackson C, Koyama S, Loebel F, Cui X, Farber SH, et al. Sequestration of T cells in bone marrow in the setting of glioblastoma and other intracranial tumors. Nat. Nat Med. 2018;24(9):1459-1468. **References 40 and 41 describe the immunosuppressive properties of the glioma microenvironment.** 10.1038/s41591-018-0135-2 PMC6129206 30104766

[42] Chiang CS, Fu SY, Wang SC, Yu CF, Chen FH, Lin CM (2012). Irradiation promotes an m2 macrophage phenotype in tumor hypoxia. Front Oncol.

[43] Leblond MM, Gérault AN, Corroyer-Dulmont A, MacKenzie ET, Petit E, Bernaudin M (2015). Hypoxia induces macrophage polarization and re-education toward an M2 phenotype in U87 and U251 glioblastoma models. Oncoimmunology.

[44] Brandes AA, Carpentier AF, Kesari S, Sepulveda-Sanchez JM, Wheeler HR, Chinot O (2016). A Phase II randomized study of galunisertib monotherapy or galunisertib plus lomustine compared with lomustine monotherapy in patients with recurrent glioblastoma. Neuro Oncol.

[45] Mangani D, Weller M, Seyed Sadr E, Willscher E, Seystahl K, Reifenberger G (2016). Limited role for transforming growth factor-β pathway activation-mediated escape from VEGF inhibition in murine glioma models. Neuro Oncol.

[46] Wang Q, He Z, Huang M, Liu T, Wang Y, Xu H (2018). Vascular niche IL-6 induces alternative macrophage activation in glioblastoma through HIF-2α. Nat Commun.

[47] Klemm F, Maas RR, Bowman RL, Kornete M, Soukup K, Nassiri S (2020). Interrogation of the microenvironmental landscape in brain tumors reveals disease-specific alterations of immune cells. Cell.

[48] Reardon DA, Brandes AA, Omuro A, Mulholland P, Lim M, Wick A (2020). Effect of nivolumab vs bevacizumab in patients with recurrent glioblastoma: the CheckMate 143 phase 3 randomized clinical trial. JAMA Oncol.

[49] Gordon SR, Maute RL, Dulken BW, Hutter G, George BM, McCracken MN (2017). PD-1 expression by tumour-associated macrophages inhibits phagocytosis and tumour immunity. Nature.

[50] Ene CI, Kreuser SA, Jung M, Zhang H, Arora S, White Moyes K (2020). Anti-PD-L1 antibody direct activation of macrophages contributes to a radiation-induced abscopal response in glioblastoma. Neuro Oncol.

[51] Wirsching HG, Arora S, Zhang H, Szulzewsky F, Cimino PJ, Quéva C (2019). Cooperation of oncolytic virotherapy with VEGF-neutralizing antibody treatment in IDH wildtype glioblastoma depends on MMP9. Neuro Oncol.

[52] Saha D, Wakimoto H, Peters CW, Antoszczyk SJ, Rabkin SD, Martuza RL (2018). Combinatorial effects of VEGFR kinase inhibitor axitinib and oncolytic virotherapy in mouse and human glioblastoma stem-like cell models. Clin Cancer Res.

[53] •• Rousso-Noori L, Mastandrea I, Talmor S, Waks T, Globerson Levin A, et al. P32-specific CAR T cells with dual antitumor and antiangiogenic therapeutic potential in gliomas Nat Commun. 2021;12(1):3615. **Interesting preclinical results on activity of P32-specific CAR T cells in glioma cells and tumor-derived endothelial cells in vitro, as well as a significant efficacy to control tumor growth in orthotopic syngeneic and xenograft mouse models.** 10.1038/s41467-021-23817-2 PMC8203650 34127674

[54] Wang Q, Hu B, Hu X, Kim H, Squatrito M, Scarpace L (2018). Tumor evolution of glioma-intrinsic gene expression subtypes associates with immunological changes in the microenvironment. Cancer Cell.

[55] Sandmann T, Bourgon R, Garcia J, Li C, Cloughesy T, Chinot OL (2015). Patients with proneural glioblastoma may derive overall survival benefit from the addition of bevacizumab to first-line radiotherapy and temozolomide: retrospective analysis of the AVAglio trial. J Clin Oncol.

[56] Johnson RM, Phillips HS, Bais C, Brennan CW, Cloughesy TF, Daemen A (2020). Development of a gene expression-based prognostic signature for IDH wild-type glioblastoma. Neuro Oncol.

[57] Lai A, Kharbanda S, Pope WB, Tran A, Solis OE, Peale F (2011). Evidence for sequenced molecular evolution of IDH1 mutant glioblastoma from a distinct cell of origin. J Clin Oncol.

[58] Unruh D, Schwarze SR, Khoury L, Thomas C, Wu M, Chen L (2016). Mutant IDH1 and thrombosis in gliomas. Acta Neuropathol.

[59] Kickingereder P, Sahm F, Radbruch A, Wick W, Heiland S, Deimling Av, et al. IDH mutation status is associated with a distinct hypoxia/angiogenesis transcriptome signature which is non-invasively predictable with rCBV imaging in human glioma Sci Rep 2015 5 16238. 10.1038/srep16238 PMC4633672 26538165

[60] Gargini R, Segura-Collar B, Herránz B, García-Escudero V, Romero-Bravo A, Núñez FJ, et al. The IDH-TAU-EGFR triad defines the neovascular landscape of diffuse gliomas Sci Transl Med 2020 Jan 22;12(527) eaax1501. 10.1126/scitranslmed.aax1501 PMC7055928 31969485

[61] Luoto S, Hermelo I, Vuorinen EM, Hannus P, Kesseli J, Nykter M (2018). Computational characterization of suppressive immune microenvironments in glioblastoma. Cancer Res.

[62] Bunse L, Pusch S, Bunse T, Sahm F, Sanghvi K, Friedrich M (2018). Suppression of antitumor T cell immunity by the oncometabolite (R)-2-hydroxyglutarate. Nat Med.

[63] Kohanbash G, Carrera DA, Shrivastav S, Ahn BJ, Jahan N, Mazor T (2017). Isocitrate dehydrogenase mutations suppress STAT1 and CD8+ T cell accumulation in gliomas. J Clin Invest.

[64] van den Bent MJ, Klein M, Smits M, Reijneveld JC, French PJ, Clement P (2018). Bevacizumab and temozolomide in patients with first recurrence of WHO grade II and III glioma, without 1p/19q co-deletion (TAVAREC): a randomised controlled phase 2 EORTC trial. Lancet Oncol.

[65] Duerinck J, Du Four S, Vandervorst F, D'Haene N, Le Mercier M, Michotte A (2016). Randomized phase II study of axitinib versus physicians best alternative choice of therapy in patients with recurrent glioblastoma. J Neurooncol.

[66] Duerinck J, Du Four S, Bouttens F, Andre C, Verschaeve V, Van Fraeyenhove F (2018). Randomized phase II trial comparing axitinib with the combination of axitinib and lomustine in patients with recurrent glioblastoma. J Neurooncol.

[67] Awada G, Ben Salama L, De Cremer J, Schwarze JK, Fischbuch L, Seynaeve L (2020). Axitinib plus avelumab in the treatment of recurrent glioblastoma: a stratified, open-label, single-center phase 2 clinical trial (GliAvAx). J Immunother Cancer.

[68] Schiff D, Desjardins A, Cloughesy T, Mikkelsen T, Glantz M, Chamberlain MC (2016). Phase 1 dose escalation trial of the safety and pharmacokinetics of cabozantinib concurrent with temozolomide and radiotherapy or temozolomide after radiotherapy in newly diagnosed patients with high-grade gliomas. Cancer.

[69] Muhic A, Poulsen HS, Sorensen M, Grunnet K, Lassen U (2013). Phase II open-label study of nintedanib in patients with recurrent glioblastoma multiforme. J Neurooncol.

[70] Norden AD, Schiff D, Ahluwalia MS, Lesser GJ, Nayak L, Lee EQ (2015). Phase II trial of triple tyrosine kinase receptor inhibitor nintedanib in recurrent high-grade gliomas. J Neurooncol.

[71] Lombardi G, De Salvo GL, Brandes AA, Eoli M, Rudà R, Faedi M (2019). Regorafenib compared with lomustine in patients with relapsed glioblastoma (REGOMA): a multicentre, open-label, randomised, controlled, phase 2 trial. Lancet Oncol.

[72] Wick W, Puduvalli VK, Chamberlain MC, van den Bent MJ, Carpentier AF, Cher LM (2010). Phase III study of enzastaurin compared with lomustine in the treatment of recurrent intracranial glioblastoma. J Clin Oncol.

[73] Weller M, Le Rhun E (2020). How did lomustine become standard of care in recurrent glioblastoma?. Cancer Treat Rev.

[74] Lombardi G, Caccese M, Padovan M, Cerretti G, Pintacuda G, Manara R (2021). Regorafenib in recurrent glioblastoma patients: a large and monocentric real-life study. Cancers (Basel).

[75] Kebir S, Rauschenbach L, Radbruch A, Lazaridis L, Schmidt T, Stoppek AK (2019). Regorafenib in patients with recurrent high-grade astrocytoma. J Cancer Res Clin Oncol.

[76] Zeiner PS, Kinzig M, Divé I, Maurer GD, Filipski K, Harter PN (2019). Regorafenib CSF penetration, efficacy, and MRI patterns in recurrent malignant glioma patients. J Clin Med.

[77] Tzaridis T, Gepfner-Tuma I, Hirsch S, Skardelly M, Bender B, Paulsen F (2019). Regorafenib in advanced high-grade glioma: a retrospective bicentric analysis. Neuro Oncol.

[78] Treiber H, von der Brelie C, Malinova V, Mielke D, Rohde V, Chapuy CI (2022). Regorafenib for recurrent high-grade glioma: a unicentric retrospective analysis of feasibility, efficacy, and toxicity. Neurosurg Rev.

[79] Werner JM, Wolf L, Tscherpel C, Bauer EK, Wollring M, Ceccon G (2022). Efficacy and tolerability of regorafenib in pretreated patients with progressive CNS grade 3 or 4 gliomas. J Neurooncol.

[80] Rudà R, Bruno F, Pellerino A, Pronello E, Palmiero R, Bertero L (2022). Regorafenib in recurrent glioblastoma: does dose reduction reduce toxicity while maintaining the efficacy?. J Neurooncol.

[81] • Shamshiripour P, Hajiahmadi F, Lotfi S, Esmaeili NR, Zare A, Akbarpour M, et al. Next-generation anti-angiogenic therapies as a future prospect for glioma immunotherapy from bench to bedside. Front Immunol. 2022;13:859633. **Updated review on underlying molecular mechanisms contributing to glioblastoma aberrant angiogenesis, clinical applications of monoclonal antibodies, tyrosine kinase inhibitors, aptamers, and small interfering RNAs as the next-generation antiangiogenic therapeutics.** 10.3389/fimmu.2022.859633 PMC9231436 35757736

[82] • Bouzari B, Mohammadi S, Bokov DO, Krasnyuk II, Hosseini-Fard SR, Hajibaba M, et al. Angioregulatory role of miRNAs and exosomal miRNAs in glioblastoma pathogenesis. Biomed Pharmacother. 2022;148:112760. **An updated review that discusses the angioregulatory effect of miRNAs and the role in GBM pathogenesis of exosomal miRNAs.** 10.1016/j.biopha.2022.112760 35228062

[83] •• Kumthekar P, Ko CH, Paunesku T, Dixit K, Sonabend AM, Bloch O, et al. A first-in-human phase 0 clinical study of RNA interference-based spherical nucleic acids in patients with recurrent glioblastoma. Sci Transl Med. 2021;13(584):eabb3945. **A first-in-human phase 0 clinical study of RNA interference–based spherical nucleic acids (SNAs) that demonstrated that SNA nanoconjugates could be a potential brain-penetrant precision medicine approach for the systemic treatment of GBM.** 10.1126/scitranslmed.abb3945 PMC8272521 33692132

[84] Saw PE, Zhang A, Nie Y, Zhang L, Xu Y, Xu X (2018). Tumor-associated fibronectin targeted liposomal nanoplatform for cyclophilin A siRNA delivery and targeted malignant glioblastoma therapy. Front Pharmacol.

[85] Ye C, Pan B, Xu H, Zhao Z, Shen J, Lu J (2019). Co-delivery of GOLPH3 siRNA and gefitinib by cationic lipid-PLGA nanoparticles improves EGFR-targeted therapy for glioma. J Mol Med (Berl).

[86] Ravi V, Madhankumar AB, Abraham T, Slagle-Webb B, Connor JR (2019). Liposomal delivery of ferritin heavy chain 1 (FTH1) siRNA in patient xenograft derived glioblastoma initiating cells suggests different sensitivities to radiation and distinct survival mechanisms. PLoS ONE.

[87] Kim SS, Harford JB, Moghe M, Rait A, Pirollo KF, Chang EH (2018). Targeted nanocomplex carrying siRNA against MALAT1 sensitizes glioblastoma to temozolomide. Nucleic Acids Res.

[88] Linder B, Weirauch U, Ewe A, Uhmann A, Seifert V, Mittelbronn M (2019). Therapeutic targeting of Stat3 using lipopolyplex nanoparticle-formulated siRNA in a syngeneic orthotopic mouse glioma model. Cancers (Basel).

